# Modified Nanodiamonds as a Means of Polymer Surface Functionalization. From Fouling Suppression to Biosensor Design

**DOI:** 10.3390/nano11112980

**Published:** 2021-11-06

**Authors:** Pavel V. Melnikov, Anastasia Yu. Alexandrovskaya, Alina O. Naumova, Nadezhda M. Popova, Boris V. Spitsyn, Nikolay K. Zaitsev, Nikolay A. Yashtulov

**Affiliations:** 1 M. V. Lomonosov Institute of Fine Chemical Technologies, MIREA—Russian Technological University, 119571 Moscow, Russia; anastasia.alexandrovskaya@gmail.com (A.Y.A.); alina.naumova.92@bk.ru (A.O.N.); nk_zaytsev@mail.ru (N.K.Z.); yashtulovna@mail.ru (N.A.Y.); 2 Federal State Unitary Enterprise Research and Technical Center of Radiation-Chemical Safety and Hygiene, Federal Medical-Biological Agency, 123182 Moscow, Russia; 3 A. N. Frumkin Institute of Physical Chemistry and Electrochemistry of the RAS, 119071 Moscow, Russia; no.hope996@gmail.com (N.M.P.); spitsyn@phyche.ac.ru (B.V.S.)

**Keywords:** detonation nanodiamond, modified nanodiamond, surface modification, fouling protection, adhesion control, fluorinated material, biofouling, oxygen sensor, optical sensor, biosensor

## Abstract

The development of different methods for tuning surface properties is currently of great interest. The presented work is devoted to the use of modified nanodiamonds to control the wetting and biological fouling of polymers using optical sensors as an example. We have shown that, depending on the type of modification and the amount of nanodiamonds, the surface of the same fluorinated polymer can have both bactericidal properties and, on the contrary, good adhesion to the biomaterial. The precise control of wetting and biofouling properties of the surface was achieved by the optimization of the modified nanodiamonds thermal anchoring conditions. In vitro and in vivo tests have shown that the fixation of amine functional groups leads to inhibition of biological activity, while the presence of a large number of polar groups of mixed composition (amide and acid chloride) promotes adhesion of the biomaterial and allows one to create a biosensor on-site. A comprehensive study made it possible to establish that in the first 5 days the observed biosensor response is provided by cells adhered to the surface due to the cell wall interaction. On the 7th day, the cells are fixed by means of the polysaccharide matrix, which provides much better retention on the surface and a noticeably greater response to substrate injections. Nevertheless, it is important to note that even 1.5 h of incubation is sufficient for the formation of the reliable bioreceptor on the surface with the modified nanodiamonds. The approach demonstrated in this work makes it possible to easily and quickly isolate the microbiome on the surface of the sensor and perform the necessary studies of its substrate specificity or resistance to toxic effects.

## 1. Introduction

Modern materials, with suitable functional properties, often have unsatisfactory characteristics in terms of adhesion, surface wettability, biocompatibility etc. One of the ways to solve this problem is modification by processing, for example, coating with functional materials [1]. As a rule, the desired characteristics are achieved by changing either the chemical structure of the material, its composition, or by changing the surface morphology. Among the most widely developed methods, one can single out processing in nonequilibrium gaseous media, in particular, in gas plasma [2].

Improvement of the mentioned surface wettability, its permeability or opposite sealability, dye absorption, improved adhesion to other materials, or protection against biofouling are attempted to be achieved by functionalizing the surface, but without changing the properties of the material in the bulk [3]. Realizing this goal, for example, in the case of polymers, is usually difficult, and it is reached using a combination of expensive procedures. Chemical and physical methods should be pointed out among the classical paths of such processing, but the expanding field of application of polymers in medicine and biomaterials requires the introduction of a new category of polymer modification based on biological methods [3].

For polymers used in food packaging, cosmetics, medicine and pharmaceuticals, biocompatibility, bactericidal properties, and biodegradability are extremely important. Immobilization of the corresponding functional coatings on the polymer surface allows one to achieve the desired properties, but the main obstacle is often insufficient wettability and poor adhesion of the layer being modified [4].

Nanodiamonds (NDs) have excellent properties such as chemical inertness, hardness and optical transparency due to the fact that these crystalline nanoparticles based on carbon inherit the crystal structure of diamond at the nanoscale [5]. They are attractive as lubricant additives, especially as they are non-toxic and biocompatible. Strict environmental standards, in particular for the content of toxic elements such as sulfur and phosphorus in lubricants, not least contribute to the introduction of ND particles as additives [6]. Another area of application of ND is medicine. For example, the use of aminated nanodiamonds has been shown to improve the mechanical properties of biomedical devices based on 3D printed resins [7]. It has been shown that polycaprolactone composites with ND additives have great potential for tissue regeneration due to the possibility of 3D scaffold extrusion. An increase in the content of ND leads to a noticeable increase in the adhesion of osteoblast tissues, in comparison with the unmodified polymer [8].

Despite the inertness of ND under normal conditions, they have different functional groups on the surface, and this opens up various paths for their modification. There are quite a few such options, and among them one can distinguish approaches of direct chemical treatment (halogenation, amination, hydrogenation, reduction and oxidation) [9,10,11,12,13] and non-covalent or covalent modification due to the addition of various molecular fragments, including the binding of biomolecules [14,15,16].

The most serious problem for the biochemical and biomedical use of carbon nanomaterials, for example, in the delivery of drugs, tissue regeneration, is toxicity [17]. A comparative study of the effect for various surface functional groups, such as –COOH, –NH_2_, –OH, showed that ND particles were cytotoxic for HEK293 cells at concentrations above 50 μg/mL. The study of the mechanism of action showed that the penetration of positively charged particles of nanodiamond through the negatively charged cell membrane leads to cytotoxicity. Moreover, carboxylated nanodiamond (ND–COOH) is embryotoxic and teratogenic [18]. In vitro antibacterial activity has also been studied. It was shown that only particles with a chemically modified surface have a significant biocidal effect. It is important to note that the type of modification has a significant effect, and aminated nanodiamonds are twice as effective as chlorinated ones. Transmission electron microscopy photographs have shown that NDs are adsorbed onto the cell surface and damage it. The authors also assume that the key is the electrostatic interaction of positively charged aminated nanoparticles with the negatively charged surface of the bacterial cytoplasmic membrane, which causes its destruction [19].

Carbon nanomaterials are actively used to create sensors, for example, for express estimation of biochemical oxygen demand (BOD) [20]. The good adhesion of the biomaterial to the working surface is the key property for such systems [21,22].

Earlier, our scientific group showed that modified nanodiamonds with various functional groups allowed for tuning of the fouling and adhesive properties of the optical sensor surface. The latter could be used to create a biosensor that can be formed onsite [23], which will allow in the future to carry out much more accurate environmental monitoring. This paper is devoted to approbation of the described approach for a real object (fish tank) and studying the conditions for the formation of biofilms on the surface for a reproducible analytical based on the readings of the optical oxygen sensor modified by means of nanodiamonds. To date, we are not aware of other works of this kind. The study consisted of two stages: in vitro and in vivo. The biofouling rate and surface properties of the sensitive elements were first evaluated with a model biological object (yeast *Saccharomyces cerevisiae*), and then a similar assessment was carried out under the conditions of a real object.

## 2. Materials and Methods

Three modifications of detonation nanodiamonds (DND) were involved as carriers of functional groups: aminated (DNDamine), chlorinated (DNDchl) and particles of mixed processing (DNDamine + chl). DNDs of the UDA-GO-SP brand (Sinta, Belarus) were used as the starting material. These are a polydisperse powders with a specific surface area of 295 m^2^/g and 30–900 nm particle size. The methodology for the DND surface modification has been described in detail earlier [24]. DNDchl were synthesized first. The pristine DNDs were heated for 5 h in Ar-atmosphere with 3% of CCl_4_ at 400 °C. The heating of DNDchl in pure ammonia for 2 h at 1 atm and 300 °C yielded DNDamine. DNDamine + chl, i.e., the product of partial conversion, was synthesized in a similar way, but the reaction time was reduced to 1 h.

The IR spectra (Bruker Equinox 55 Fourier transform IR spectrometer (Bruker Optik, Ettlingen, Germany) with a diffuse reflectance accessory PIKE Technologies Eas-iDiffTM) were used to assess the presence of functional groups in the samples by comparing them with the spectrum of the initial DND. The actual particle size distribution was determined using LS 13 320 XR Particle Size Analyzer (Beckman Coulter, Brea, CA, USA).

The optical oxygen sensor composite material served as the modifiable substrate. Its structure and the process of properties optimization have been described earlier [25,26]. The material consists of two phases, each one performs its own function. The first phase is formed by nanostructured SiO_2_ microparticles (Merck silica gel 60), which serve as adsorption centers for the indicator dye. The latter was represented by the complex of Pt (II) 5,10,15,20-tetrakis (2,3,4,5,6-pentafluorophenyl)-porphyrin (PtTFPP, Frontier Scientific, www.frontiersci.com (accessed on 4 October 2021)), which is widely used both in commercial oxygen analyzers and in scientific research [27]. The second phase is represented by the fluorine-containing polymer, fluoroplastic 42 (F42, HaloPolymer, www.halopolymer.ru (accessed on 4 October 2021)), which serves as the media for microparticles distribution and protects the sensor from both dye leaching even in an environment containing hydrocarbons and from biological fouling [28,29]. A similar approach could be used in sensors for other substances [30].

The process of nanodiamonds anchorage to the surface layer has been studied in detail earlier [31]. DNDs were placed in glycerol before applying to the sensor surface being modified (round films with an average diameter of 1 cm). The suspension was kept for 20 min in an ultrasonic bath, and then 30 μL was immediately applied to the samples. Annealing was carried out in a drying oven (SNOL-3,5,3,5,3,5/3,5-I2M, Thermix, Moscow, Russia) at the temperature of 160 °C in an argon atmosphere for 12 h.

Preliminary research has shown that the highest biofouling was revealed for the sample with DNDamine + chl (4.6 × 10^−4^ g·cm^−2^). This kind of coating is suitable for the in vivo bioreceptor element formation [23]. DNDamine (2.3 × 10^−4^ g·cm^−2^), on the contrary, reveals inhibition of fouling, thus it could be used in an environment with a high biomass content as a reference sensor. DNDchl also exhibits fouling inhibition, but to a much lesser extent compared to DNDamine, and therefore we did not use it in this work. Samples with the indicated content of modified DNDs were used, and, accordingly, suspensions of 6 and 12 g·dm^−3^ were prepared to obtain samples of sensor material with DNDamine and DNDamine + chl. Quanta 650 FEG scanning electron microscope (FEI Company, Hillsboro, OR, USA) was used for the sensor surface electron micrographs acquisition.

**Figure 1:**
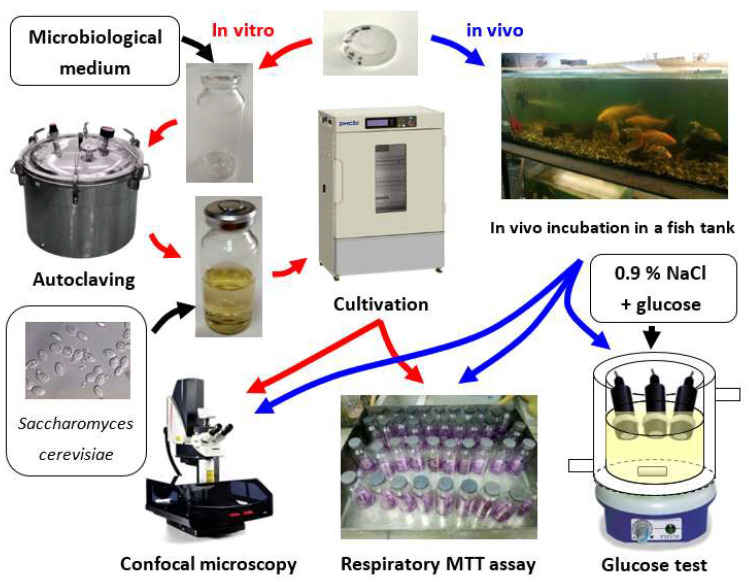
General scheme of experiments.

In vitro and in vivo experiments with three types of sensitive elements were performed according to the scheme in Figure 1: unmodified composite material F42 (as reference), sample with 2.3 × 10^−4^ g·cm^−2^ DNDamine and sample with 4.6 × 10^−4^ g·cm^−2^ DNDamine + chl. The biofouling rate and the surface properties of the sensitive elements were estimated in the first experiment; the second one was devoted to the assessment under the real object conditions (fish tank).

Pure cultures of the yeast *Saccharomyces cerevisiae* were used as a model biological object in this work. They rapidly build up biomass and readily form biofilms [32]. In vitro cultivation in a liquid microbiological medium was carried out for 1.5 h, 3, 5 and 7 days at 28 °C. The composition was as follows (g·dm^−3^): K_2_HPO_4_, 0.655; NH_4_Cl, 1.0; MgSO_4_ × 7H_2_O, 0.2; FeSO_4_ × 7H_2_O 0.01; CaCl_2_ 0.0075; Sucrose 50.0. Pre-sterilization of the medium was carried out for 40 min at 110 °C and 1.5 bar using an autoclave. Four replications of each measurement were used to assess the reproducibility. Absorbance at 660 nm was used for biomass growth estimation (SF 2000 spectrophotometer, OKB Spectr, Saint-Petersburg, Russia). Goryaev chamber at 1200 magnification served for visual cell counting.

The colorimetric MTT assay was used for the estimation of biofilm formation on the sensitive elements’ surfaces [33,34]. This method allows one to establish the viability of cells by assessing the cellular metabolic activity by the ability to reduce the yellow salt of 3-(4,5-dimethylthiazol-2-yl)-2,5-diphenyltetrazolium bromide (MTT). Laser confocal scanning microscopy (Leica SP5 microscope (Leica, Wetzlar, Germany)) made it possible to visualize the biofilm. Cells were stained by fluorescent dye SYTO^®^ 11 (S7573 ThermoFisher, Walthamcity, MA, USA) diluted 1:1000 in phosphate buffer. Lectin IV from wheat germ agglutinin (WGA) conjugated with fluorescent dye Alexa Fluor 488 (W11261 ThermoFisher, Waltham, MA, USA) was used for polysaccharide matrix staining [35]. The Nomarski contrast method allowed for detecting undyed particles. An argon laser with a wavelength of 488 nm (for detecting WGA fluorescence) and 594 nm (for detecting SYTO 11) was used for images acquisition, followed by digital analysis with Imaris 7.0.0 software package (Bitplane, Zürich, Switzerland).

During the in vivo experiment samples were placed for 1.5 h, 3, 5 and 7 days into the 250 L fish tank with the following species: *Carassius auratus*, *Dawkinsia arulius*, *Barbus jae*, *Barbus schwanenfeldi* and other species of the *Cyprinidae* family. The temperature range was 18–23 °C. Fish feeds from the manufacturers Tetra, Dajana and Sera were used. The incubation was followed by biofilm formation assessment. Respiratory MTT assay and confocal scanning microscopy were used in the same way as in the in vitro experiment.

In addition, a glucose test (served as model substrate) was performed to assess the biosensory capabilities of the modified materials. Expert-009 optical oxygen analyzer (Econix-Expert, Moscow, Russia) was used for these measurements. The design and operation principles were described earlier [36]. The decay time τ of the phosphorescence of the indicator dye is calculated from the phase shift φ of the measured analytical signal relative to the modulated exciting light according to Equation (1):

τ = tan (φ)/2π*f*, (1)

where *f* represents the modulation frequency. Oxygen saturation and dissolved concentration are then calculated using a calibration plot. The samples were attached to oxygen analyzers and immersed in 50 mL of saline solution with stirring at 28 °C, then glucose was added stepwise and the readings were evaluated.

## 3. Results

### 3.1. Material Properties

**Figure 2:**
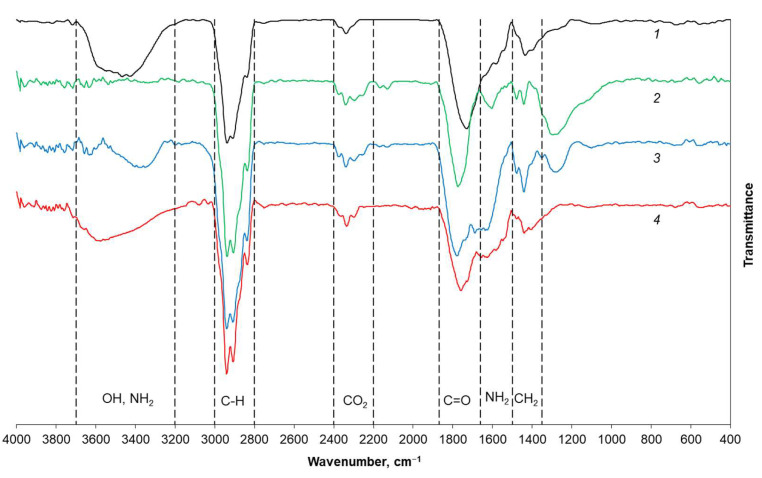
IR Fourier spectra of DND samples: (1) pristine DND, (2) DNDchl, (3) DNDamine, and (4) DNDamine + chl [31].

The DND surface contains different functional groups, and their type, amount, and mutual ratio are directly determined by the method of particle synthesis [37]. The most convenient determination of the existing functional groups can be carried out using IR spectroscopy [31]. The spectra of chemically modified DND samples were compared with the initial UDA-GO-SP spectrum (Figure 2). The spectrum of the pristine DND contains several characteristic peaks: C=O stretching vibrations of the carbonyl group (maximum ~1720 cm^−1^), which is a part of the carboxyl group, typical valence (3000–2800 cm^−1^) and torsion (maximum 1440 cm^−1^) peaks of C–H vibrations, and a wide band of O–H stretching vibrations (3700–3200 cm^−1^). The latter can be both a part of a carboxyl group and a group on the DND surface [38]. In addition, one can see the band of CO_2_ present in the air (2400–2200 cm^−1^).

Noticeable differences are observed in the spectrum of DNDchl obtained at the first stage of chemical modification. A new broad band appears in the 1000–1300 cm^−1^ region. Its unambiguous correlation is difficult, since it is in the “fingerprint” area. Such absorption can be observed, for example, in terminal alkyl halides, where the C–H vibration of the –CH_2_X group is observed in the range 1300–1150 cm^−1^. The O–H group peak completely disappears, and a shift of the C=O band is observed (maximum ~1780 cm^−1^). The latter can be explained by a change in the local environment, for example, by the proximity to the Cl atom. Thus, it can be said that acid chloride (R−COCl) groups have formed on the surface. The spectrum also changes after the conversion of DNDchl to DNDamine: a peak appears again in the region of 3700–3200 cm^−1^, which now corresponds to stretching N−H vibrations, and a previously absent intense peak in the region of 1620–1670 cm^−1^ is visible. It corresponds to the band characteristic of all secondary amides, which arises due to the shift in the frequency of stretching vibrations of C=O group. They are shifted due to the proximity to the more electronegative atom N to the high-frequency region and to the low-frequency region due to resonance with a hydrogen bond and the lone electron pair of N atom [38]. It is also worth noting a significant decrease in the intensity of the band in the 1000–1300 cm^−1^ region. DNDamine + chl, which are the partial conversion particles, also exhibit the characteristic band of amides (1620–1670 cm^−1^), but the intensity is almost two times lower. That indicates the partial substitution of chlorine atoms by NH_2_ groups, as well as the presence of both types of functional groups on the surface.

**Figure 3:**
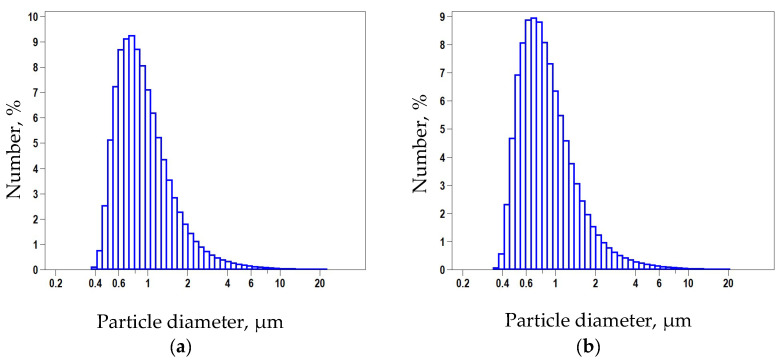
Average size distribution measured by dynamic light scattering: (**a**) pristine DND; (**b**) DNDamine.

Measurement of the actual particle size distribution was carried out to control possible aggregation during chemical surface modification. Comparison of the samples before and after functionalization did not reveal significant differences (Figure 3), and this is expected, since the modification involves only the surface layer, without affecting the actual particle size. Mean value 876 nm, median 799 nm, which matches the manufacturer’s specification. 

**Figure 4:**
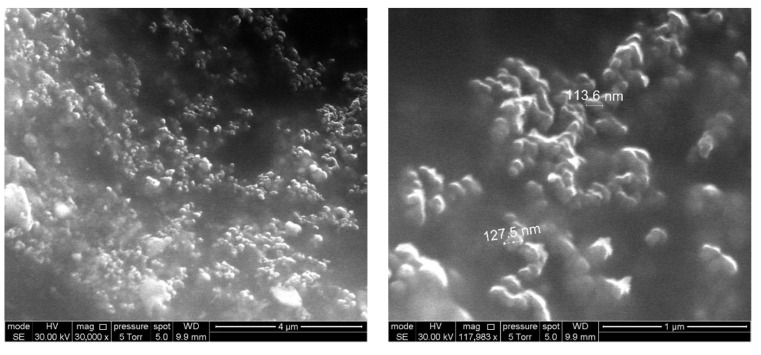
Examples of SEM images of DNDamine coating with a nanodiamond content of 2.3 × 10^−4^ g·cm^−2^ on the surface of polymer F42.

Modified nanodiamonds were anchored in the upper layer of the polymer F42, which served as the matrix for optical oxygen sensor. The surface of the used polymer softens at the temperature of 160 °C, and therefore it is optimal for embedding. The micrographs of the sensor surface obtained using scanning electron microscopy (SEM) show nanodiamond particles fused into the structure of an amorphous polymer (Figure 4).

### 3.2. In Vitro Experiment

The formation and fixation of the extracellular polysaccharide matrix can be used to assess the susceptibility of a material to microbial contamination, since it is this matrix that promotes cell adhesion to the surface. The latter is extremely important for creating a stable biosensor, but, on the contrary, is harmful when measuring the true oxygen content in a biomass medium. During the in vitro experiment, the rate of biofilm formation on the materials modified with DND was assessed.

**Figure 5:**
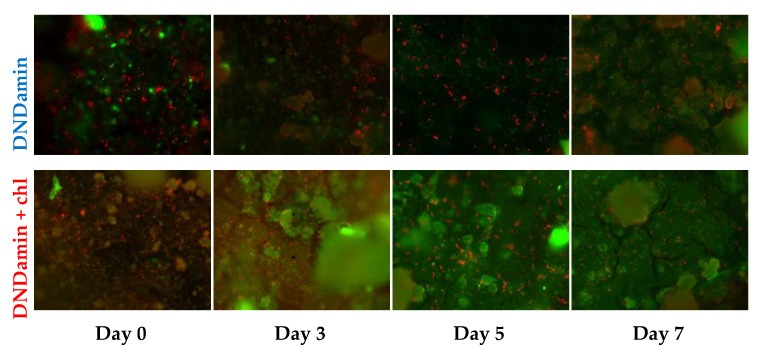
Images of modified polymers samples (1200×) after incubation in medium with *Saccharomyces cerevisiae* yeast at 28 °C for various times and fluorescent staining. The cells are shown in red, the polysaccharide biofilm matrix is indicated in green.

Photographs obtained by laser confocal scanning microscopy are shown in Figure 5. They clearly show an increase in the area of the polysaccharide matrix with an increase in the incubation time in a microbiological environment. The rate is obviously superior in the case of DNDamine + chl coating.

**Figure 6:**
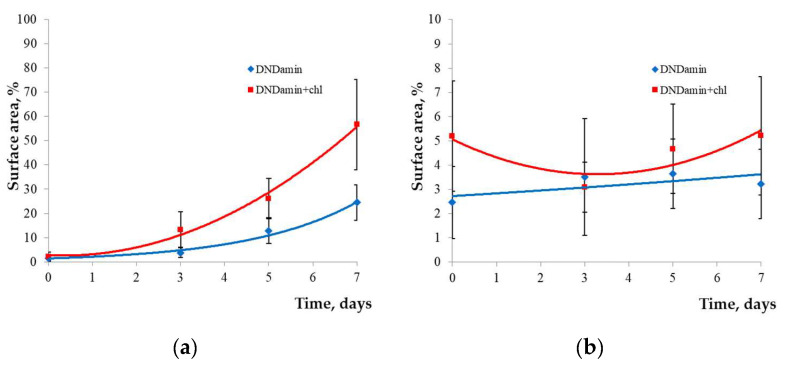
Processing of confocal microscopy images of samples modified with DNDamine and DNDamine + chl. Change over time of the calculated areas occupied by: (**a**) the polysaccharide matrix; (**b**) *Saccharomyces cerevisiae* cells.

Processing with specialized software made it possible to quantify the percentage of surface area occupied by the biofilm and cells (Figure 6). The growth of the biofilm area during the studied period of the week occurs exponentially, but the rates of fouling of samples with different DND modifications differ significantly. By the 7th day, the average occupied area for DNDamine + chl is 56.7% versus 24.5% for DNDamine. At the same time, the cells count found on the surface practically does not change with time (Figure 6b), but the amount is also noticeably higher in the case of DNDamine + chl samples.

**Figure 7:**
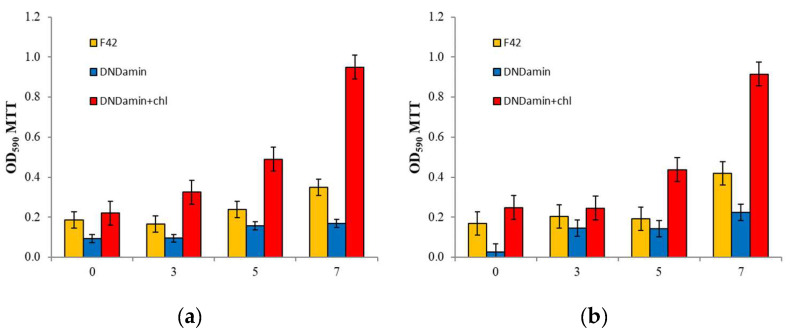
Respiratory activity of cells on the samples (MTT assay optical density) after 1.5 h, 3, 5 and 7 days: (**a**) In vitro incubation with culture of the *Saccharomyces cerevisiae*; (**b**) in vivo incubation in a fish tank.

The differences in materials properties revealed by the results of confocal microscopy, nevertheless, do not allow one to estimate the physiological state of cells fixed on the surface. To assess this criterion, the MTT assay was used, which makes it possible to determine the respiratory activity of the formed films (Figure 7a). Significant growth is observed for DNDamine + chl with increasing incubation time, and the dependence is similar to Figure 6a. Thus, the increase in the area of the polysaccharide matrix correlates with the count of the living cells on the surface of this material.

The dependence is fundamentally different for the sample coated with DNDamine. The living cells count changes little over time, while it is always less than that found on untreated F42 material. Thus, it can be stated that the DNDamine coating demonstrates inhibition of fouling, and a substantial part of the cells found on the material surface is dead. The results obtained agree with the study of the antibacterial activity of non-immobilized modified nanodiamonds in vitro [19]. The estimated reproducibility (deviation from the mean) is natural for microbiological measurements [33,34].

### 3.3. In Vivo Experiment

This experiment was designed to evaluate the change in the characteristics of materials with different DND coatings under the conditions of a real object (fish tank). In general, the differences in the rate of biofilm formation revealed at the preliminary in vitro stage were confirmed. It is possible to compare the results of the respiratory MTT assay in Figure 7 as confirmation. Small differences are observed within the margin of error of the method, but general trends persist in the transition from a model to a real object.

**Figure 8:**
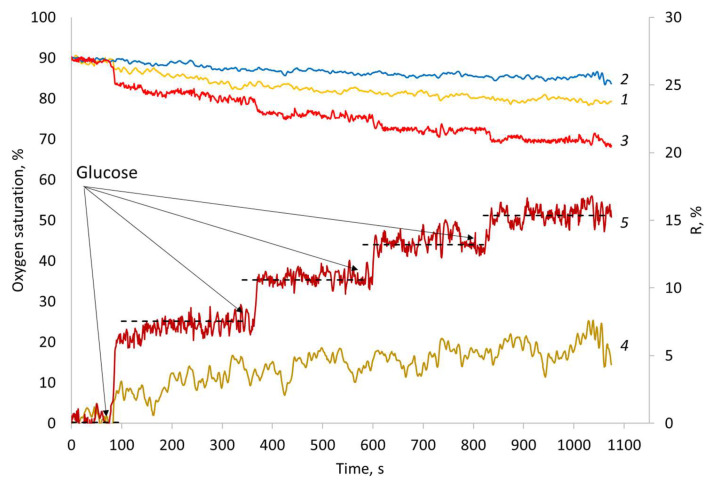
Sensors responses to glucose injections after 1.5 h in vivo incubation (left axis) for: (1) unmodified material F42; (2) DNDamine; (3) DNDamine + chl. Calculated analytical signal R for (4) unmodified material F42 and (5) DNDamine + chl.

Figure 8 shows the time dependence of the readings of sensors with different types of coverage. It can be seen that the curves for all three sensors differ, and the deviation increases with the introduction of glucose into the system. The readings of the DNDamine coated sensor show only a very small decrease in the measured oxygen concentration and do not change with the addition of glucose into the solution. Wherein the DNDamine + chl coated sensor always reveals lower measured concentration, and responds to stepwise glucose additions. Such a difference may occur due to the respiratory activity of cells adsorbed on the surface of the DNDamine + chl sensor; as a result, the analyzer detects a local decrease in the oxygen concentration.

Thus, the biological response R can be represented as the difference between the readings of analyzers with different coatings. Such series were calculated for couples (DNAamin − F42) and (DNAamine − DNAamine + chl) (Figure 8, series 4 and 5). Obviously, the reaction of the DNDmin + chl coated sensor is much higher than that observed for the unmodified F42 material. In addition, the steps caused by the introduction of glucose are clearly visible on it. The observed differences are associated with an increase in the wettability of the surface with applied DNDamine + chl and the fixation of a larger number of respiring cells on it, which is confirmed by the MTT assay (Figure 7b).

**Figure 9:**
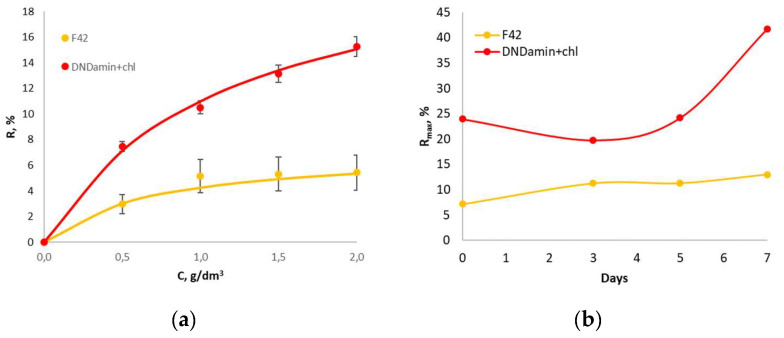
(**a**) Dependence of the analytical signal R on the glucose concentration for unmodified material F42 and DNDamine + chl; (**b**) Dependence of the R_max_ value on the incubation time.

The calibration dependence of the response R of the untreated and DND modified materials on the glucose concentration is shown in Figure 9a. Since the whole cells receptor elements are actually the catalytic type bioreceptors [39,40], the dependencies are fit by the following Michaelis–Menten equation:

R = R_max_[S]/(K_M′_ + [S]), (2)

where R_max_ denotes the maximal oxygen uptake rate achieved by the immobilized microorganisms at substrate concentration [S] → ∞ and K_M′_ is the apparent Michaelis constant, meaning the substrate concentration at which R = R_max_/2. For the dependencies in Figure 9a, the parameters of the equation are: R_max_—7.20 and 23.95; K_M′_—0.70 and 1.18 for untreated material and DNDamine + chl modified material, respectively.

We traced the evolution of the R_max_ parameter with the time of in vivo incubation (Figure 9b). A slight almost linear growth is observed for the untreated material, while a significant increase is witnessed for the DNDamine + chl coating on the 7th day of incubation. Comparison with the results of confocal microscopy and respiratory MTT assay allows us to conclude that in the first 5 days the observed response is provided by cells adhered to the sensor surface due to the cell wall interaction. On the 7th day, the cells are fixed by means of the polysaccharide matrix, which provides much better retention on the surface and a noticeably greater response to glucose injections. Nevertheless, it is important to note that even 1.5 h of incubation is sufficient for the formation of a bioreceptor on the surface with the modified DND with pronounced reactions to the substrate.

## 4. Discussion

It is obvious that the type and the number of functional groups on the surface of the studied materials are the main reasons for the differences observed. An increase in the number of polar groups introduced due to the anchoring of modified DNDs leads to an enhancement in wettability and a significant increase in biofouling. It is important to note that despite the fact that it is the effective adhesion of DND particles that is the reason of their antibacterial [19] and antiviral [41] activity in solution, immobilization leads to a limitation of the cytotoxic effect. In other words, the surface of the same polymer can have both bactericidal properties and, conversely, good adhesion to the biomaterial, depending on the type of modification and the amount of nanodiamonds. This work not only demonstrates this phenomenon, but also shows an example of its application - the creation of a biosensor directly at the analysis site (fish tank). It is important to note that the analytical response of the system to the substrate injections is represented not by the readings of one sensor layer, as in traditionally used microbial biosensors [20,21,22], but by the difference between the readings of two technically identical sensors, differing only by the type and amount of nanodiamonds anchored to surface. The coating with DNDamine demonstrates the suppression of fouling, and such a sensor actually shows the oxygen concentration in the bulk volume, while the coating with DNDamine+chl determines the local oxygen depletion in the biofilm layer.

The observed effect of inhibition of biofouling in the case of DNDamine coating seems to have the same mechanism as that observed for polycationic polymers — guanidine derivatives, which are known antiseptic agents [42,43,44]. In the case of the latter, close contact of a substance with a high density of positively charged groups with cell walls violates the integrity of cell membranes and leads to death [19]. Similar effects are observed with other carbon-based nanoparticles that have been widely investigated for bio and medicine applications [45]. One of the key mediators of nanomaterials-induced inflammation could be activation of NLRP3 inflammasome [46]. In particular, it was found that nanodiamonds can disrupt lysosomal membrane and subsequently activate NLRP3 via cathepsin B activation pathway [47]. The lack of cytotoxic activity of DNDamine + chl coatings can be explained by the presence of two types of functional groups with different polarities on the surface of nanodiamonds. The mutual compensation of their charges occurs, and the required critical density is not reached. In this case, the surface wettability is remarkably improved, which in turn leads to a noticeable increase in biological fouling [31].

It is important to point out that the modification affects only the thinnest surface layer of the product, and the properties of the bulk part of the material are not distorted. In particular, the calibration dependence for an optical sensor practically does not change, which is extremely important for practical use.

It is known that the structure and activity of the microbial community can change in response to environmental conditions. This ability to reflect the external influences could be used to create microsensors, and such systems have great potential for practical use [48]. An actively growing area is the application of biosensor technologies, together with integrated machine learning algorithms, that allow the collection of huge amounts of data. Such systems are the future of microbiome analysis, and they can be used, in particular, in a clinical setting for more accurate and rapid diagnosis [49,50]. The approach demonstrated in this work makes it possible to easily and quickly isolate the microbiome on the surface of the sensor and perform the necessary studies of its substrate specificity or resistance to toxic effects. DND modified sensor substrates could be integrated into microfluidic systems with flow injection not only to increase their sensitivity and reproducibility, but also to create new methods for rapid environmental monitoring, based not on such averaged indicators as BOD, but on assessing the susceptibility of a particular microbiome to certain external influences.

## Data Availability

The data presented in this study are available on request from the corresponding author.
